# Linking metabolic dysfunction with cardiovascular diseases: Brn-3b/POU4F2 transcription factor in cardiometabolic tissues in health and disease

**DOI:** 10.1038/s41419-021-03551-9

**Published:** 2021-03-12

**Authors:** Vishwanie S. Budhram-Mahadeo, Matthew R. Solomons, Eeshan A. O. Mahadeo-Heads

**Affiliations:** 1 Molecular Biology Development and Disease, Institute of Cardiovascular Science, University College London, London, UK; 2 College of Medicine and Health, University of Exeter Medical School, St Luke’s Campus, Exeter, UK

**Keywords:** Cardiovascular diseases, Diabetes

## Abstract

Metabolic and cardiovascular diseases are highly prevalent and chronic conditions that are closely linked by complex molecular and pathological changes. Such adverse effects often arise from changes in the expression of genes that control essential cellular functions, but the factors that drive such effects are not fully understood. Since tissue-specific transcription factors control the expression of multiple genes, which affect cell fate under different conditions, then identifying such regulators can provide valuable insight into the molecular basis of such diseases. This review explores emerging evidence that supports novel and important roles for the POU4F2/Brn-3b transcription factor (TF) in controlling cellular genes that regulate cardiometabolic function. Brn-3b is expressed in insulin-responsive metabolic tissues (e.g. skeletal muscle and adipose tissue) and is important for normal function because constitutive Brn-3b-knockout (KO) mice develop profound metabolic dysfunction (hyperglycaemia; insulin resistance). Brn-3b is highly expressed in the developing hearts, with lower levels in adult hearts. However, Brn-3b is re-expressed in adult cardiomyocytes following haemodynamic stress or injury and is necessary for adaptive cardiac responses, particularly in male hearts, because male Brn-3b KO mice develop adverse remodelling and reduced cardiac function. As a TF, Brn-3b regulates the expression of multiple target genes, including GLUT4, GSK3β, sonic hedgehog (SHH), cyclin D1 and CDK4, which have known functions in controlling metabolic processes but also participate in cardiac responses to stress or injury. Therefore, loss of Brn-3b and the resultant alterations in the expression of such genes could potentially provide the link between metabolic dysfunctions with adverse cardiovascular responses, which is seen in Brn-3b KO mutants. Since the loss of Brn-3b is associated with obesity, type II diabetes (T2DM) and altered cardiac responses to stress, this regulator may provide a new and important link for understanding how pathological changes arise in such endemic diseases.

## Facts

- Indisputable links exist between common metabolic diseases such as obesity and type II diabetes and increased risk of heart diseases.
- Molecular regulators driving these changes are not well-understood.
- Emerging evidence identifies important functions for the Brn-3b/POU4F2 transcription factor in controlling homoeostasis in metabolic and cardiovascular tissues.
- Reduction/loss of Brn-3b causes metabolic dysfunction, e.g. profound hyperglycaemia and insulin resistance, as well as abnormal responses to haemodynamic stress.

## Open questions

- What are the roles of Brn-3b/POU4F2 target genes in controlling normal function in insulin-responsive tissues and the heart?
- How does the loss of Brn and its target genes contribute to metabolic dysfunction and abnormal cardiac responses?
- Are cardiac abnormalities in male Brn-3b KO caused by direct or indirect effects in cardiac cells?

## Introduction

Cardiovascular diseases (CVDs) remain the main cause of mortality globally, accounting for an estimated 31% of deaths worldwide^1,2^. CVDs are set to rise significantly, in line with a global obesity epidemic because metabolic diseases such as obesity and type 2 diabetes mellitus (T2DM) are known risk factors for CVDs^3–5^. Thus, obesity strongly correlates with increased risk of adverse cardiovascular events, including atherosclerosis, cardiac arrhythmias and heart failure^6–8^. Similarly, T2DM is recognised as an independent risk factor for heart disease since patients with T2DM display a higher incidence of MI with poorer outcomes compared with non-diabetic patients^8–10^. Despite strong evidence supporting close links between such diverse diseases, the molecular mechanisms driving such changes are still to be elucidated.

Physiological adaptation and pathological changes arise due to alterations in genes that control key cellular processes^11,12^ so identifying factors that control gene expression is important for understanding the disease process. Chronic diseases such as obesity, type II diabetes and CVD, are particularly challenging because while gene expression changes may occur at early stages of the disease, the pathological changes often develop over relatively long periods. Therefore, symptoms become detectable at later stages, when irreversible tissue damage has already occurred^9^, thereby limiting effective treatment options and increasing the burden on population health and healthcare^13^. Thus, elucidating the molecular mechanisms that drive such pathological changes will improve our understanding of these diseases but also facilitate early diagnosis/treatment, aimed at minimising or reversing pathological changes before irreversible damage occurs.

Since pathological changes that drive chronic diseases are caused by altered gene expression^11^, then identifying the tissue-specific transcription factors that regulate such cellular genes will provide insight into the mechanisms that drive pathological changes associated with common diseases^14–17^. This review focuses on novel roles for the transcription factor, Brn-3b/POU4F2 (called Brn-3b) in maintaining metabolic and cardiac function.

## Brn-3b/POU4F2 transcription factor (TF)

**Fig. 1:**
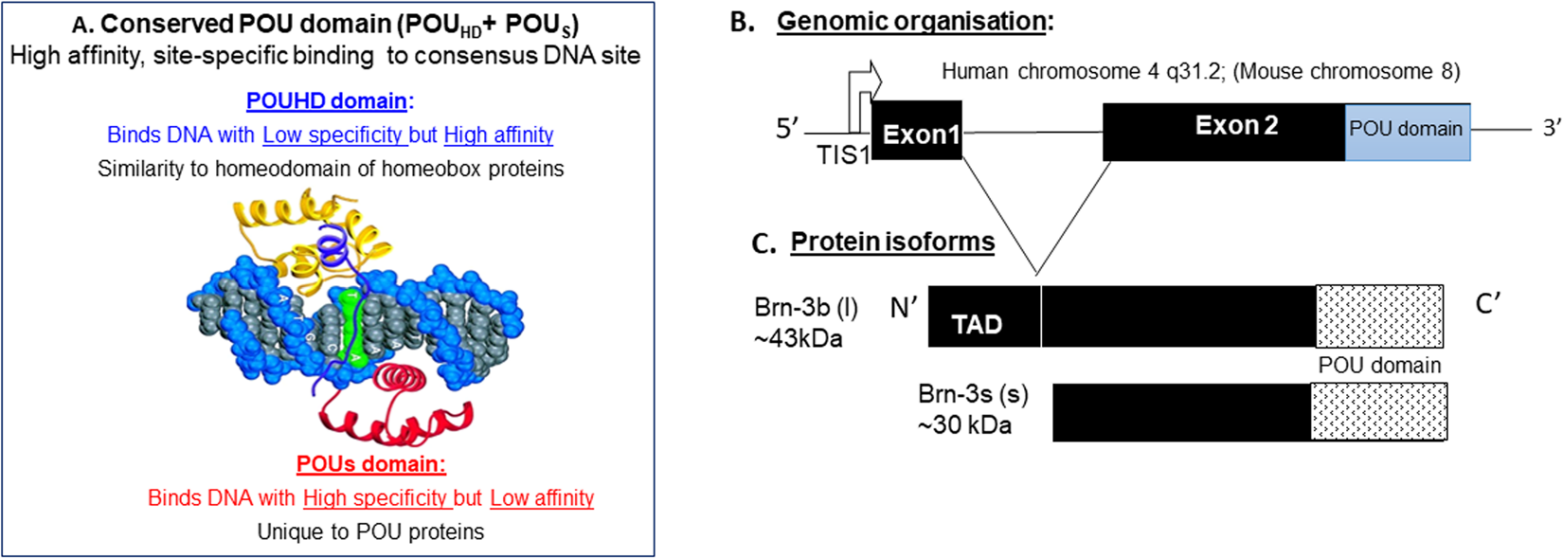
Schematic figures showing the structure of the POU domain, genomic organisation of the Brn-3b gene and proteins arising from this gene. **A** Diagram showing a schematic model of the DNA-binding POU domain in Brn-3b TF, which is unique to the POU family of transcription factors. This domain consists of the POU_HD_ which binds to the major groove of the DNA double helix with high affinity but low specificity while the POU-specific domain (POUs), which is unique to POU proteins, binds to DNA with high specificity but low affinity. The POUs and POU_HD_ domains are tethered together by a poorly conserved linker and together these domains confer high specificity and high-affinity DNA binding to sites in gene promoters. POU_HD_ POU homeodomain, POU_S_ POU-specific domain. **B** Schematic diagram showing the genomic organisation of the Brn-3b locus on human chromosome 4 (mouse chromosome 8). **C** Schematic diagram showing Brn-3b protein isoforms, with splicing of exons 1 and 2 giving rise to the longer Brn-3b(l) while the shorter Brn-3b(s) protein is encoded by exon 2 and therefore contains the POU domain but lacks the amino-terminal transactivation domain (TAD) found in the Brn-3b (l). Approximate sizes of the proteins are indicated. TIS transcription initiation site, TAD transactivation domain, N’ = amino terminal, C’ carboxy terminal.

Brn-3b is a tissue-specific TF^18–25^ belonging to the class IV POU (*Pit-1; Oct-1; Unc-86*) sub-family of homeodomain TFs, and is characterised by a highly conserved POU domain that forms a helix–turn–helix (HTH) DNA binding structure^26^. The POU domain consists of a 60aa homeodomain, POU-_HD_, with strong similarity to homeobox proteins and 74–82aa POU-specific domain (POU_s_), which is unique to POU proteins (Fig. 1A). The POU_HD_ and POU_S_ domains are tethered by a poorly conserved linker and act together to facilitate high-affinity site-specific binding to A/T-rich DNA sequences in the promoters of target genes, thereby regulating the rate of gene transcription^27,28^.

Brn-3b was originally isolated from proliferating neuronal derived cell line ND7, due to its high homology to the POU domain of the related Brn-3a protein^29^. However, these proteins are encoded by distinct genes on different chromosome, i.e. Brn-3b: human chr 4q31.2 (mouse chr 8) and Brn-3a: human chr 13q31.1 (mouse chr 14)^30,31^. The Brn-3b gene consists of two exons separated by a single intron (Fig. 1B) and can give rise to two distinct protein isoforms, i.e. Brn-3b(s), encoded by exon 2 only and the longer Brn-3b(l) isoform, produced by splicing of exons 1 and 2. Consequently, Brn-3b(l) contains an N’ trans-activation domain encoded by exon 1, not found in Brn-3b(s) (Fig. 1C). However, both isoforms contain the DNA-binding POU domain, encoded by exon 2, so can bind to the BRNF DNA consensus site to regulate (activate or repress) tissue-specific target genes^30,31^. In addition, a feedback mechanism facilitates auto-regulation by these isoforms to control protein levels^32–34^. At the amino acid level, Brn-3b is highly conserved across diverse species (Table 1), suggesting important functions in determining cell fate/function^28^.

**Table 1:** Comparison of Brn-3b amino acid homology between human and rodents (mouse and rat) and zebrafish.

Species	% Homology to human Brn-3b
Mouse	97.3%
Rat	97.3%
Zebrafish	87%

**Fig. 2:**
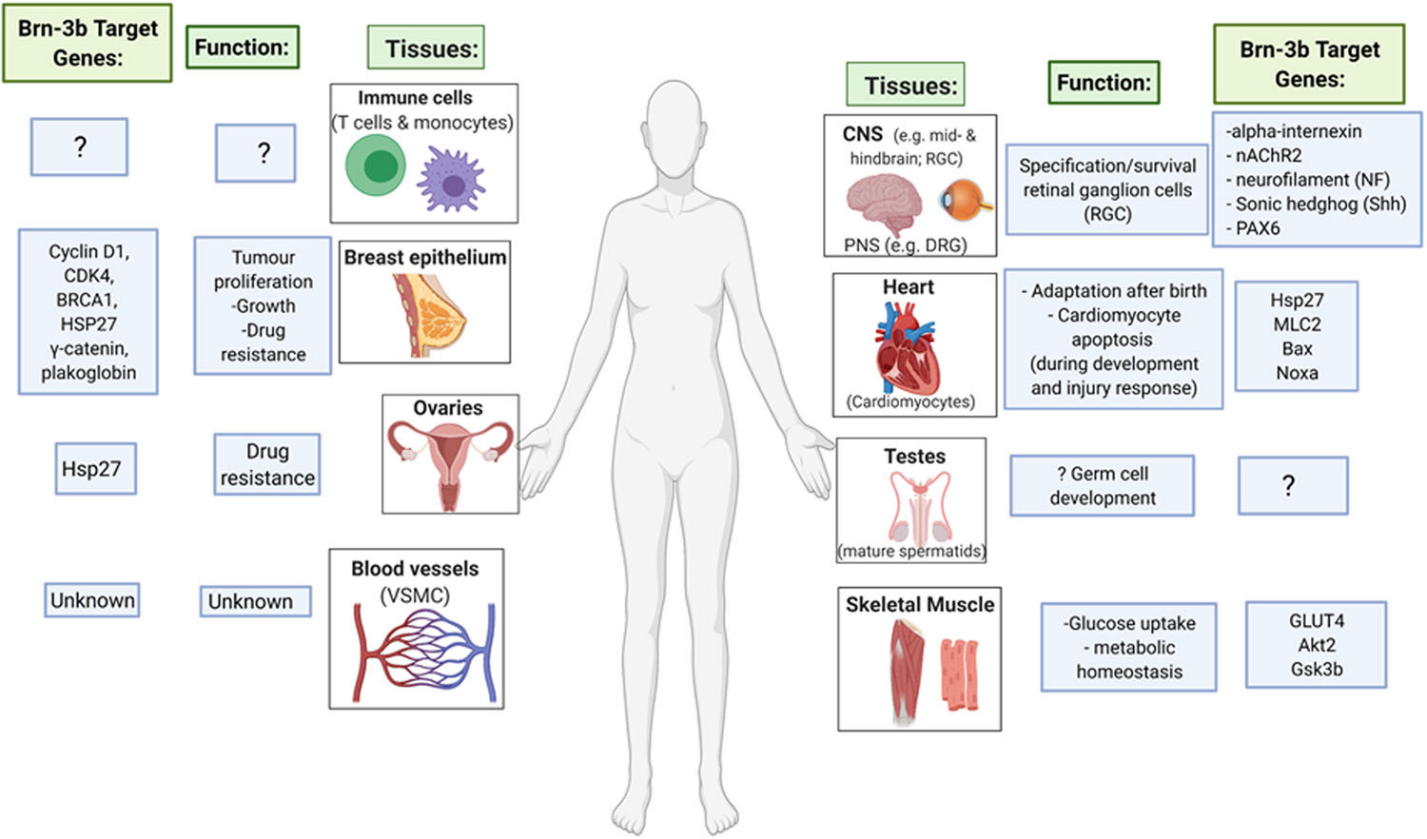
Representation of different tissues that express Brn-3b. The effects of Brn-3b in different tissues and target genes, where known, are also listed.

Since Brn-3b was isolated from neuronal cells, early studies were focused on its expression in the central nervous system (CNS), e.g. midbrain, hindbrain and retinal ganglion cells (RGC)^25,35,36^ and peripheral nervous system (PNS), e.g. afferents of dorsal root ganglia (DRG); cranial nerves V, VIII, IX, X and VII^37^. However, subsequent studies have shown Brn-3b expression in other diverse tissues including reproductive tract tissue (testis, ovary and breast epithelium)^22,33,34,38–44^, peripheral blood mononuclear cells (PBMC)^23,45,46^, metabolic tissue (adipocytes, skeletal muscle and liver)^20^, cardiovascular tissues e.g. cardiomyocytes^18,19,21,39,47^ and vascular smooth muscle cells (VSMC) (Fig. 2). Moreover, early studies using constitutive Brn-3b knockout (KO) mice revealed essential roles for Brn-3b in maintaining survival and specification of retinal ganglion cells (RGCs), since homozygous Brn-3b KO mutants are blind, due to loss of ~70% of RGCs, post-natally^24,25,30,35^. More recent studies using these mutants have also highlighted essential and previously unknown roles for Brn-3b in regulating genes that control the fate and function of metabolic, vascular and cardiac cells^18,20^ (see later).

**Table 2:** List of known Brn-3b target genes with selected cellular functions and potential effects of changes on clinical conditions.

Target genes	Selected cellular function	Effects of altered gene expression
*Genes directly activated by Brn-3b*		
Cyclin D1 and CDK4	Cell cycle progression/proliferation, inhibit gluconeogenesis in metabolic tissues; increased expression in stressed heart	Increase associated with cancer; loss or reductions linked to abnormal cardiac responses to stress, T2DM
GLUT 4	Glucose uptake/metabolism; insulin responsiveness	Reduced expression linked to insulin resistance in T2DM Loss or reduction in stressed hearts
HSP-27	Cell survival, actin polymerisation, cell motility	Drug resistance/migration in cancers Cardioprotective in stressed hearts
SHH	Morphogen with pleotropic effects	RGC development; heart development, coronary revascularisation
nACHr2	Neurotransmitter; activation of neuromuscular junction	
*Genes repressed by Brn-3b*		
α-internexin	Intermediate filament	
BRCA1	Cell cycle inhibitor, apoptosis	Reduced expression or loss of function in cancers
Plakoglobin	Adhesion molecule, adherence junction,	Reduced expression in cancers
GSK3β	Pleotropic protein kinase, controls glycogen synthesis and glucose metabolism; negative regulator of hypertrophy	Increased expression in T2DM, heart disease
*Genes indirectly regulated by Brn-3b (upon interaction with other regulators)*		
Bax; Noxa; Puma (Brn-3b + p53)	Apoptosis	Heart failure
Hsp27 (Brn-3b + ER)	Survival, growth and cell motility	Drug resistance; migration in cancer cells Cardioprotective in stressed hearts

## Brn-3b controls gene transcription directly or indirectly by interaction with other regulators

As a transcription factor Brn-3b can drive complex cellular effects, including proliferation^42,43,48^, differentiation, survival^49,50^ and metabolic processes^20^ by controlling the expression of tissue- or signal-specific target genes, depending on cell types and growth conditions (see Table 2).

**Fig. 3:**
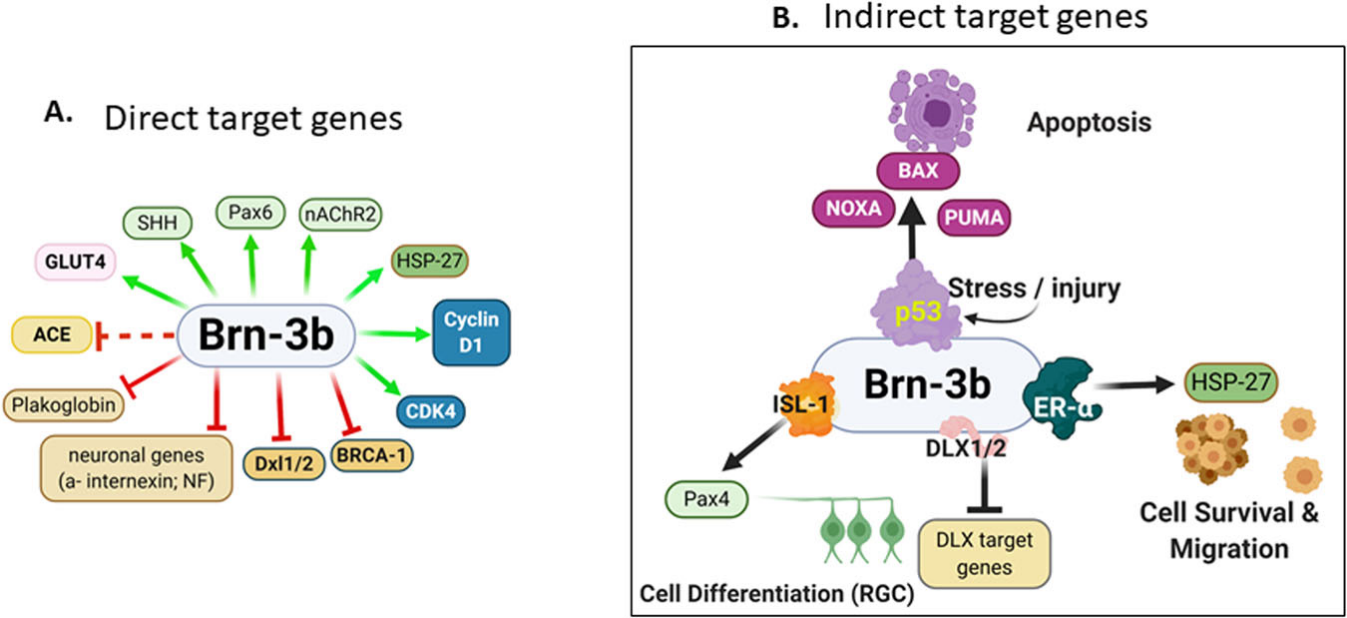
Schematic diagram showing the effects of Brn-3b on known target genes. **A** Representation of known target genes that are directly regulated by Brn-3b. Green arrows indicate genes that are directly activated activated by Brn-3b and red bars represent genes that are repressed. **B** Shows genes that are indirectly regulated by Brn-3b, upon interaction with other cellular proteins, including the oestrogen receptor alpha (ER-α), p53 tumour suppressor gene; ISL1 and DLX proteins, as indicated. The effects on cell fate are also indicated.

Direct effects can arise from Brn-3b binding to BRNF-binding sites, on target gene promoters or enhancers and either activating or repressing transcription of target genes (Table 1 and Fig. 3A). For example, in neuronal cells, Brn-3b activates the nicotinic acetylcholine receptor 2 (nAChR2) subunit promoter^51^ but represses α-internexin^52^ and neurofilament^53^. However, in RGCs, Brn-3b activates other genes including PAX6, sonic hedgehog (SHH) but represses DLX1/2^54–56^. In proliferating cells (e.g. breast cancers), Brn-3b can increase cell division^43,48^ by activating promoters of genes encoding cell cycle proteins cyclin D1^40^ and CDK4^42^, while repressing the tumour suppressor BRCA1^44^. Brn-3b also confers drug resistance and migratory potential in cancer cells by indirectly activating heat shock protein 27 (Hsp27)^34,39^, while repressing adhesion molecule, plakoglobin (gamma catenin)^41^. As such, elevated Brn-3b is associated with growth, drug resistance and migratory potential in different cancers^34,43,44,48,57^; Brn-3b is also implicated in the regulation of metabolic genes that control glucose uptake, e.g. GLUT4 and GSK3β^20^ (later).

However, Brn-3b can control gene expression indirectly by interacting with other cellular proteins to regulate other target genes. For instance, in RGCs, Brn-3b interacts with Isl-1 and DLX 1/2 to control RGC gene expression during retinal development^50,58,59^. Other notable Brn-3b interacting partners, oestrogen receptor (ER)^60^ and p53 tumour suppressor protein^61^ are discussed below to highlight the complex mechanisms by which Brn-3b controls cell-specific or condition-specific effects (Fig. 3B).

## Brn-3b interaction with ERα

Brn-3b interaction with ERα was originally identified and characterised in epithelial-derived breast cancer cells, which express both proteins^60,62^. In these cells, Brn-3b interacts and cooperates with ERα to maximally transactivate ERE containing promoters e.g. Hsp27 (Fig. 3)^34,60^. As a target gene, Hsp27 is particularly important because of its diverse effects on cell function, including inhibition of apoptosis and regulation of cytoskeletal dynamics, e.g., actin polymerisation^63–65^. Consequently, maximal activation of Hsp27 by co-expression of Brn-3b and ER may promote cell survival, drug resistance and metastatic potential in cancer cells^66–69^.

However, like Brn-3b, Hsp27 is required for normal heart development and is also implicated in cardioprotection in stressed adult hearts^70–73^. Since ER proteins are also expressed in the heart, vasculature and metabolic tissues^74–79^, it is likely that co-expression of Brn-3b and ER in these tissues may also regulate relevant target genes. Therefore, it is important to analyse if/how co-expression of Brn-3b and ER may also affect gene expression and cell fate in cardiometabolic tissues.

## Brn-3b interaction with p53 controls apoptosis

Brn-3b also interacts with p53 protein^61^ and when co-expressed, Brn-3b cooperates with p53 to increase the transcription of pro-apoptotic target genes e.g. *Bax* and *Noxa*, thereby promoting cell death through the mitochondrial pathway (Fig. 3B and Table 1)^47,61^. In fact, the Brn-3b:p53 interaction may be necessary for maximal induction of apoptotic genes, since primary neuronal cells prepared from Brn-3b KO mutants express reduced pro-apoptotic *Bax* and are resistant to apoptosis-inducing staurosporine, despite p53 expression being unchanged^61^.

The cooperation between Brn-3b and p53 to enhance apoptosis has been pivotal for elucidating how cell fate decisions are determined under different conditions. For example, while Brn-3b can promote cell proliferation and growth by activating cell cycle genes such as CDK4 and cyclin D1^40,80^, induction of p53 in Brn-3b expressing cells (e.g. by stress or injury) can cause conflicting signals and trigger a mitotic crisis^61,81^. This is because p53 can inhibit cell growth by inducing cell cycle inhibitors e.g. p*21*^*cip1/waf1*^^82,83^ or promote apoptosis by activating apoptotic genes such as *Bax, Noxa and Puma*^84,85^. Under such conditions, cooperation between Brn-3b and p53 to synergistically activate pro-apoptotic genes and trigger apoptosis. provides a paradigm to explain how cell fate is determined in the face of conflicting signals^86^ and also highlights the complex mechanisms by which Brn-3b drives diverse effects on cell fate (Fig. 3). This interaction between Brn-3b and p53 is also important in controlling the gene expression and fate of cardiomyocytes, in both developing and adult hearts^18,57^. The mechanisms associated with controlling effects in cardiomyocytes will be discussed in more detail later, but it is noteworthy that ectopic increases in Brn-3b in the developing heart can induce apoptosis if co-expressed with p53 and thereby contribute to congenital heart defects^19^. Similarly, Brn-3b is increased in cardiomyocytes of stressed or injured adult hearts and is required for the maximal induction of pro-apoptotic genes by p53 in the infarct zone following coronary artery ligation^18^ (described below). Therefore, the effects of Brn-3b on gene expression and cell fate must be considered in the context of cell type as well as growth conditions that induce other co-regulators.

## Brn-3b in metabolic and cardiovascular tissues

The remainder of this review will focus on Brn-3b in metabolic and cardiovascular tissues, where it has a novel and potentially important roles in controlling gene expression and cell fate.

## Brn-3b in metabolic tissues

Recent studies have shown that Brn-3b is expressed in insulin-responsive metabolic tissues, including skeletal muscle, liver and adipose tissues^20^ and in vitro studies using myoblast- derived C2C12 cells have confirmed that Brn-3b is, itself, regulated by key metabolites and pathways that control metabolic processes since Brn-3b mRNA and protein are increased by glucose but inhibited by insulin^20^. Furthermore, compelling evidence from Brn-3b KO mice supports essential roles for Brn-3b in controlling important metabolic processes since KO mutants develop increased body weight when compared to WT, despite no significant differences in food intake. Glucose-tolerance studies have shown that these mutants develop profound hyperglycaemia and insulin resistance characteristic of T2DM following glucose bolus administration^20^. Importantly, Brn-3b is also significantly reduced in tissues taken from normal mice fed on high-fat diet to induce obesity, and such reduction correlates with the development of hyperglycaemia and insulin resistance. Since the loss of Brn-3b (KO mutants) or reduced expression (obese mice) is linked to marked metabolic dysfunction, these data indicate important roles for Brn-3b in controlling metabolic processes^20^.

Potential metabolic target genes regulated by Brn-3b in skeletal muscle have been identified using mRNA from Brn-3b KO tissues and matched WT controls that was used to screen a disease-focused RT^2^ Profiler PCR Array (containing 84 metabolic genes), followed by qRT-PCR to confirm changes. Noteworthy target genes that are reduced upon loss of Brn-3b include the insulin-dependent glucose transporter, GLUT4 and angiotensin 1-converting enzyme (ACE1), while the glycogen-synthase kinase 3-beta, (GSK3β) was significantly increased in tissues from Brn-3b KO mice^20^. Based on the known functions of these genes, it is likely that such changes will contribute to the abnormal metabolic phenotype seen in Brn-3b KO mutants. For example, GLUT4 plays a central role in insulin-mediated glucose uptake in diverse tissues^87^ and reduction of this transporter e.g. in GLUT4^+/−^ mice is associated with peripheral insulin resistance, similar to that seen in Brn-3b KO mutants^88^. Co-transfection studies and ChIP assays confirm that Brn-3b can directly activate the GLUT4 gene promoter while significant reduction of GLUT4 mRNA and protein levels in skeletal muscle and adipose tissues taken from Brn-3b KO mice^20^ confirm that Brn-3b is important for regulating GLUT4 expression. Therefore, Brn-3b regulation of such target genes will be controlling normal metabolic processes in insulin-responsive tissues.

In contrast, increased expression of GSK3β in Brn-3b KO tissues suggests that Brn-3b may normally repress this enzyme and therefore loss of Brn-3b will cause corresponding increases in GSK3β. In metabolic tissues, GSK3β controls glycogen synthesis by phosphorylating and inactivating glycogen synthase^89^, and is also implicated in controlling glucose metabolism by its effects on other metabolic enzymes such as glucose-6-phosphate and its receptors e.g. insulin receptor, IRS1^90,91^. Accordingly, increased GSK3β in metabolic tissues is implicated in diseases such as T2DM and elevated expression has been reported in skeletal muscle taken from diabetic patients with insulin resistance^89–91^. Therefore, increased GSK3β upon loss of Brn-3b may also contribute to metabolic dysfunction in Brn-3b KO mutants.

Other known Brn-3b target genes such as cyclin D1 and CDK4 are also implicated in controlling glucose metabolism, independently of cell cycle regulation. In this context, activation of cyclinD1-CDK4 can cause inhibition of gluconeogenesis by regulating the activity of GCN5 histone acetyltransferase and thereby inhibiting PGC1α, a regulator of mitochondrial biogenesis^92^. Although not present on the PCR array used for screening, reduced cyclin D1 and CDK4 upon loss of Brn-3b could also contribute to metabolic dysfunction in Brn-3b KO mutants.

## Brn-3b in the developing heart

Brn-3b is expressed in cardiomyocytes of the developing heart^19,21^ and although found at low levels in adult hearts under normal conditions, it is strongly re-expressed following stress and injury^18,47^. This is similar to other foetal cardiac TFs such as NKX2.5, HAND2 and Brn-3a, which are re-expressed in adult hearts following stress/injury and are important for driving cellular responses to injury^47,93,94^. Therefore, studying the roles of such regulators during the development of the mature four-chambered heart^95,96^ can provide insight into potential mechanisms of action in stressed adult hearts^97^.

In the developing heart, Brn-3b has essential but partially redundant roles with the related Brn-3a TF, with which it shares >95% homology in the POU domain^57^ so can drive compensatory transcriptional effects on some target genes e.g. activation of Hsp27^21^. However, differences outside the POU domain may also cause antagonistic effects on other target genes and elicit opposing effects on cell fate^47,52^. For example, while Brn-3b cooperates with p53 to maximally activate apoptotic genes and increase cell death, Brn-3a blocks p53 activation of pro-apoptotic genes but co-operates with p53 to increase the cell cycle inhibitor, p21^cip1/Waf1^, thereby promoting survival and differentiation^47,61,98,99^.

Importantly, Brn-3b is significantly increased in hearts from Brn-3a KO mutants and can partially compensate for the loss of Brn-3a, because double KO (Brn-3a^−/−^: Brn-3b^−/−^) mutants are embryonic lethal and die by E8.5 (i.e. before cardiac looping)^19,100^. However, it was possible to use zebrafish (ZF) embryos as a model for studying Brn-3a and Brn-3b during early heart development because both proteins are highly conserved (>80%) between ZF and mammals (Table 1) and are expressed in ZF heart by 48 h post fertilisation (HPF)^19^. Studies using ZF embryos have provided evidence for essential roles for Brn-3a and Brn-3b in controlling normal heart development since reducing both proteins, using morpholinos, is sufficient to block cardiac looping^19^, an essential stage in heart morphogenesis^95,101^.

Although increased Brn-3b may partially compensate for Brn-3a loss in the developing heart, ectopic increases in Brn-3b and resultant increases in its target genes e.g. cyclin D1 correlate with hyperproliferation in heart valves, ventricular wall and septum in E14.5 Brn-3a KO mutant hearts^21^. At later stages, induction of p53, when combined with such increased Brn-3b results in cooperative activation of pro-apoptotic genes, *Bax, Noxa*; *Puma* and increased cell death. Therefore, by E18.5, Brn-3a KO hearts display cardiomyocyte death, reduced myocardial compaction, hyper-trabeculation and myocardial fissures, which may contribute to the death of mutants shortly after birth^19,102,103^.

The molecular mechanisms underlying the compensatory effects between Brn-3b and Brn-3a in the heart are still being investigated, but are likely to result from regulation of common target genes e.g. HSP27, which contributes to cardiomyocyte differentiation and adaptation to stress in either the developing heart or in stressed adult hearts^21,72,104,105^. Moreover, this study also shows how the complex interplay between Brn-3b and other regulators can control cardiomyocyte fate during normal heart development and may also be pertinent to stress responses in adult hearts.

### Brn-3b in adult hearts following acute injury or chronic stress

As previously mentioned, Brn-3b is found at relatively low levels in normal adult hearts but is significantly upregulated in cardiomyocytes following myocardial infarction (MI) or haemodynamic stress e.g. pressure/volume overload^19,47^.

In the context of acute injury, Brn-3b mRNA is rapidly induced in adult rat hearts, following coronary artery ligation. This model, which induces ischaemia/hypoxia and is commonly used to mimic MI, causes rapid increases in Brn-3b mRNA by 1 h post injury that continues to increase, with maximal expression at 24 h. Levels remain above baseline at 1-week post ligation^47^. Detailed localisation studies in heart sections have confirmed that Brn-3b is increased throughout the injured heart, with expression being detected in cardiomyocytes around the infarct zone as well as in the remote, uninjured myocardium.

Cellular processes in the myocardium can vary significantly depending on the location in relation to the site of injury. Therefore, Brn-3b expression throughout the injured heart reflects potentially complex effects for this TF in regulating cellular responses at different locations in the injured myocardium. This is highlighted by analysis of Brn-3b co-localisation with p53, in the reported study^47^ showing that while Brn-3b is expressed throughout the heart, p53 is only detected in cardiomyocytes around the infarct zone. Importantly, co-localisation of p53 with Brn-3b in injured cells correlates with the induction of pro-apoptotic genes (e.g. *Bax, Noxa*, *Puma)* and associated enhanced apoptosis^19,47^. Indeed, studies carried out using primary rat ventricular cardiomyocyte cultures (NRVC) indicate that Brn-3b cooperation with p53 is required for the maximal induction of pro-apoptotic genes such as *Bax* and *Noxa*, following ischaemic or hypoxic injury. Thus, short interfering RNA (siRNA) to reduce endogenous Brn-3b expression in NRVM cultures is sufficient to attenuate *Bax* and *Noxa* expression following ischaemia/reoxygenation injury, despite p53 expression remaining unchanged^47^. Therefore, co-expression of Brn-3b with p53 may be required for maximal induction of pro-apoptotic genes and effective clearance of non-viable cardiomyocytes in the infarct zone.

While the reported effects have focused on the cooperation between Brn-3b and p53 in activating pro-apoptotic gene expression and cell fate in injured cardiomyocytes within the infarct zone, the effects of increased Brn-3b in the remote, uninjured myocardium (which lacks p53 and shows no evidence of apoptosis), remains to be elucidated^47^. Under such conditions, cardiomyocytes in the remote, uninjured myocardium also undergo extensive hypertrophic adaptation to compensate for the increased workload caused by the loss of contractile myocardium. However, the factors that control genes required for such adaptive responses in the remote myocardium are still not fully understood^106^. It is therefore intriguing that recent evidence has highlighted essential roles for Brn-3b in controlling adaptive responses in hypertrophic hearts (see below)^18^. Although speculative, it is possible that Brn-3b may control adaptive responses in remote myocardium and thereby help to maintain cardiac output and function of the injured heart.

### Brn-3b in adaptive hypertrophic responses following chronic haemodynamic stress

More recently, Brn-3b has been implicated in previously unknown roles for controlling adaptive hypertrophic responses in adult hearts, following sustained haemodynamic stress that increases cardiac workload^18^. Cardiac hypertrophy occurs in response to increased workload on the heart because terminally differentiated cardiomyocytes have limited proliferative capacity, and therefore undergo hypertrophy to maintain cardiac output^107^. Such responses are characterised by extensive genetic reprogramming, cytoskeletal reorganisation and metabolic adaptation depending on the type and duration of stress^108^. For example, increased physiological demands (pregnancy or endurance exercise) cause reversible hypertrophic responses^109^ while chronic haemodynamic stresses (pressure or volume overload), can trigger a complex, biphasic response in which early adaptive responses are necessary for maintaining cardiac output/function^110^, while sustained stresses trigger maladaptive changes, including cardiomyocyte apoptosis and interstitial fibrosis and remodelling; which underpins the pathogenesis of heart failure^111,112^. These distinct responses are driven by gene expression changes and are associated with different cellular responses. For instance, adaptive hypertrophic responses are associated with re-expression of foetal genes, including natriuretic peptides, (ANP/BNP), which help to normalise wall stress by modulating myocyte growth and fibroblast proliferation^113,114^; β-MHC, which is linked to sarcomeric reorganisation and increased metabolic genes e.g. GLUT4 to facilitate metabolic switching^115–121^. In contrast, pathological changes are associated with increased expression of pro-apoptotic genes (e.g. p53 and Bax) that drives cardiomyocyte apoptosis and pro-fibrotic regulators e.g. TGF-β signalling pathways, linked to fibrosis and remodelling^110^. However, the molecular mechanisms that control the transition from adaptive to maladaptive responses are not fully understood. Therefore, identifying transcription factors that control genes required for adaptive hypertrophic responses in the heart will be important for controlling functional outcomes in stressed hearts^107^.

In this regard, Brn-3b is a potential contender since in-vivo and in-vitro studies show that this TF is strongly activated by known hypertrophic stimuli, including angiotensin II (AngII), endothelin and calcineurin A (CnA), either in intact hearts or in isolated primary cardiomyocyte cultures^18^. Detailed analysis of the Brn-3b promoter also shows that activation by AngII occurs via MAPK/ERK1 signalling while CnA acts via NFAT activation and the convergence of such distinct signalling pathways in activating the Brn-3b promoter suggests that this regulator plays a central role in controlling hypertrophic responses in the heart^18,107^.

The roles of Brn-3b as a key regulator of cardiac hypertrophy are also supported by the fact that Brn-3b and its target genes, GLUT4 and cyclinD1 are increased in hypertrophic cardiomyocytes expressing β-MHC and showing characteristic sarcomeric reorganisation^18^. Furthermore, studies carried out using Brn-3b KO mice, showed that loss of Brn-3b causes attenuated hypertrophic responses to AngII treatment, as confirmed using multiple endpoints, including heart weight:body weight (HW:BW) ratio, LV mass, cardiomyocyte cell volume and lack of hypertrophic marker, β-MHC, when compared with controls^18^. Importantly, loss of Brn-3b also affects heart function in male mice, as assessed using echocardiography and pressure–volume loop analysis. Thus, hearts from male Brn-3b KO mice display adverse functional changes, including reduced cardiac output (CO) and ejection fraction (EF), but increased end-systolic volume (ESV). This correlates with histological changes such as increased extracellular matrix (ECM) deposition, extensive fibrosis and pathological remodelling in the LV wall and around the coronary artery when compared with appropriate controls^18^. At the molecular level, Brn-3b target genes e.g. GLUT4 are significantly reduced male in Brn-3b KO hearts and may contribute to the maladaptive cardiac responses following AngII treatment. These data suggest that Brn-3b is necessary for the male heart to undergo adaptive hypertrophic changes following haemodynamic stress and loss of Brn-3b may contribute to maladaptive remodelling associated with progression to heart failure.

### Brn-3b: linking metabolic dysfunction and CVD

The predicted global increase in heart disease is strongly influenced by the current surge in metabolic diseases, such as obesity and T2DM^1,122–124^. Therefore, elucidating the molecular mechanisms that link such diverse systemic changes in cardiovascular and metabolic systems will be useful for understanding the disease processes but can also enable earlier intervention to prevent or reverse adverse changes before the onset of irreversible pathology that underlies heart diseases^125^.

Data emerging from recent studies have shown that reduction or loss of Brn-3b and its target genes are linked to metabolic dysfunctions as well as maladaptive cardiac responses and may therefore help to understand the molecular basis linking metabolic diseases and CVDs^18,33^. Many observations that link the loss of Brn-3b with metabolic dysfunction and adverse cardiac responses to stress arise from studies using the global Brn-3b KO mice . However, these are strongly supported by additional data using either wild-type mice or in-vitro models^18–20,47^. For example, while the first evidence showing an association between a loss of Brn-3b and metabolic dysfunction (hyperglycaemia and insulin resistance) has been identified using constitutive Brn-3b KO mutants in vitro studies have confirmed that Brn-3b, is itself, regulated by key metabolites such as glucose, insulin and fatty acids, which are dysregulated in obesity and T2D^20^. Moreover, in-vivo studies carried out using wild-type mice show that diet-induced obesity, which causes hypoglycaemia insulin resistance also correlated with significant reduction of Brn-3b in metabolic tissues^20^.

Similarly, in-vivo models have been pivotal for demonstrating that Brn-3b is induced in stressed adult hearts e.g. following AngII infusion or coronary artery ligation in WT mouse and rat models and is supported by using in vitro cultures of isolated primary cardiomyocytes to confirm Brn-3b induction, primarily in cardiomyocytes. However, the requirement for Brn-3b in controlling adaptive responses is convincingly demonstrated from studies in the constitutive Brn-3b KO mutants, which display attenuated hypertrophic responses to AngII treatment, when compared with responses in WT controls. The adverse cardiac responses in male Brn-3b KO hearts have also provided invaluable evidence to support the essential roles for Brn-3b in driving adaptive responses to stress.

As a TF, Brn-3b can mediate diverse effects by controlling the expression of different target genes in a tissue- or condition-specific manner. Identification of GLUT4 and GSK3β as potential Brn-3b target genes in metabolic tissues such as skeletal muscle and adipose tissue suggests important roles for this regulator in controlling metabolic processes. However, these genes are also expressed in the heart, so it is important to consider how changes in expression may affect such diverse tissues. For instance, in metabolic tissues, GLUT4 potentiates insulin-mediated glucose uptake and reduced GLUT4 levels correlate with hyperglycaemia and insulin resistance in insulin-responsive tissues, as seen in Brn-3b KO mice. However, this glucose transporter is also relevant in the stressed heart when metabolic switching (i.e. fatty acid oxidation to glucose utilisation) occurs as part of the adaptive hypertrophic response. Accordingly, reduced GLUT4 expression in Brn-3b KO hearts may contribute to the adverse responses to stress^121,126^.

Similarly, GSK3β is a key regulator of glucose homoeostasis and increased expression in metabolic tissues is strongly associated with insulin resistance. Therefore, increased GSK3β expression in Brn-3b KO tissues suggest that Brn-3b may normally repress GSK3β and loss of Brn-3b will result in increased expression in mutant tissue, thereby contributing to metabolic dysfunction^20^. However, GSK3β is also a negative regulator of cardiac hypertrophy in adult hearts^89,90,127^ and increased expression in the heart is associated with attenuated hypertrophic responses^128,129^, also observed in Brn-3b KO mutant hearts following AngII treatment. GSK3β is also activated in the heart during ischaemic injury and has been implicated in adverse post-MI remodelling since GSK3β KOs show preserved LV function following injury^130^. It will be important to elucidate the mechanism by which this TF controls GSK3β expression in cardiomyocytes to understand how Brn-3b contributes to adaptive responses and why the loss of Brn-3b causes maladaptive responses in stressed adult hearts.

It is also important to note that the initial screening for Brn-3b target genes in metabolic tissues was done using a highly targeted approach of screening PCR arrays with 84 selected metabolic genes. However, this array did not include many known Brn-3b target genes including sonic hedgehog (*shh*), cyclin D1 and CDK4, which are implicated in controlling metabolic processes and/or cardiac responses, under different conditions^92^. For example, the pleiotropic morphogen SHH was identified as a potential Brn-3b target gene in RGC^54,131^, but is also essential for normal heart development^132^ and cardiac responses to injury. Thus, SHH activation in the heart, following ischaemia-reperfusion injury, confers cardioprotective effects on the coronary vasculature and cardiomyocyte contractility^133^ and its inhibition is associated with increased infarct size and poor cardiac function following MI^134^ and poor cardiac function in diabetic mouse hearts^135^. Therefore, it is important to determine if Brn-3b regulates SHH expression in the injured heart because then loss of Brn-3b and reduced SHH may contribute to abnormal cardiac responses following stress/injury in Brn-3b KO hearts. Similarly, Brn-3b target genes, cyclinD1 and CDK4, which normally control cell cycle progression, are also implicated in controlling gluconeogenesis by blocking PGC1α^92^. CyclinD1 is also increased in adult hearts following chronic stress or injury but its role in hypertrophic responses is not fully understood^18^. Therefore, the reduction of such Brn-3b target genes may also contribute to the adverse effects seen in Brn-3b KO hearts.

**Fig. 4:**
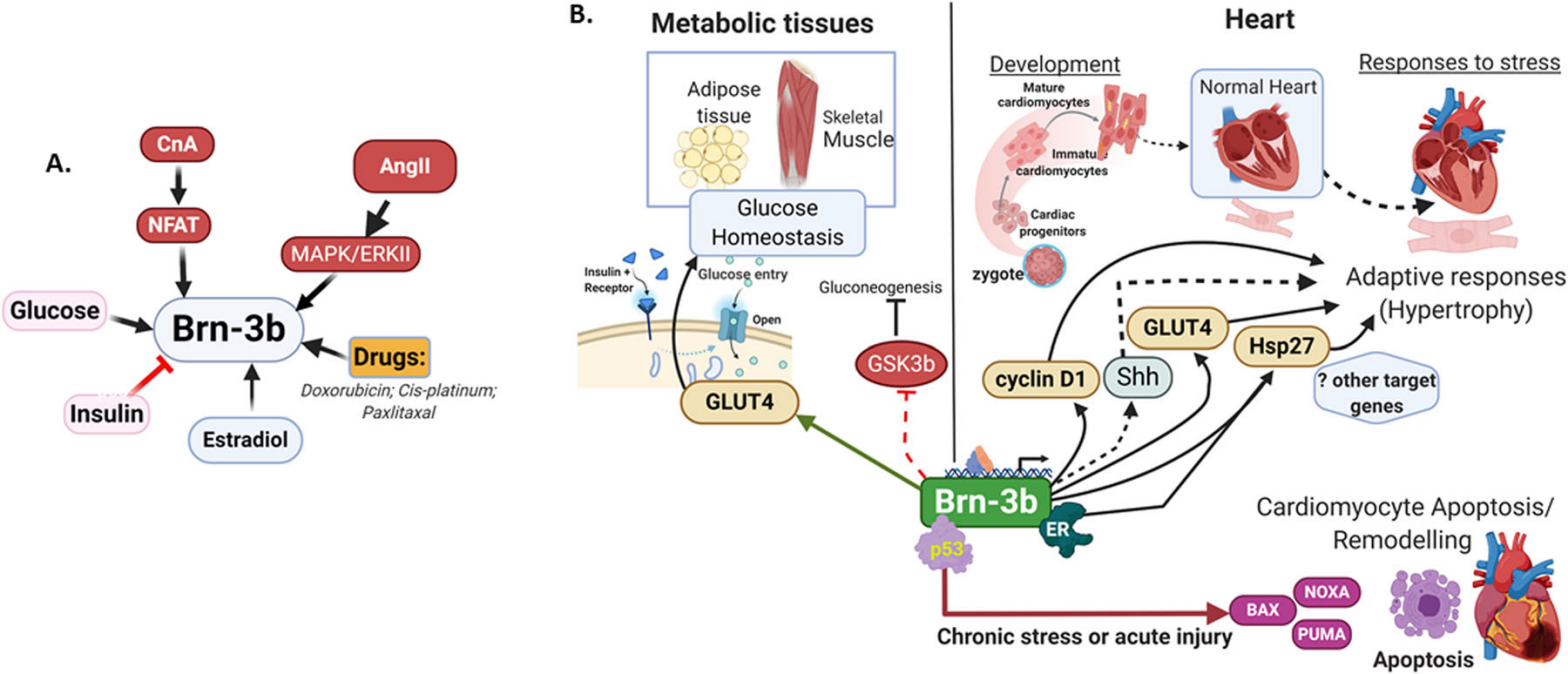
Schematic diagram showing factors that regulate Brn-3b expression and tissue-specific target genes that it may control in cardiac and metabolic tissues. **A** Schematic diagram showing growth effectors and signalling pathways that are known to regulate Brn-3b expression. Black arrows indicate activators; red bars indicate inhibitory effects. **B** Schematic diagram showing the proposed effects by which Brn-3b and its target genes can give rise to functional effects in either metabolic tissues or in the heart. High levels of Brn-3b in the foetal heart are reduced in normal adult hearts but significantly re-expressed in response to stress. Brn-3b target genes are associated with adaptive hypertrophic responses in the absence of p53. However, chronic stress or acute injury that induces p53 in Brn-3b expressing cells will cause cooperative activation of pro-apoptotic target genes, associated with cardiomyocyte apoptosis and remodelling. Solid arrows indicate genes that are directly regulated by Brn-3b while dotted arrows indicate proposed effects (activation or repression).

The co-existence of metabolic and cardiovascular dysfunctions in the constitutive Brn-3b KO mutants combined with these findings support Brn-3b TF as an important regulator of metabolic processes and adaptive cardiac responses to stress, especially in male hearts (Fig. 4). While this study has focused on how the loss of Brn-3b affects male hearts, it will also be important to study the cardiac responses of female KO hearts to stress. This is important because Brn-3b and ER can cooperate to regulate key target genes while oestrogens and ERs have known beneficial cardiac effects, particularly in pre-menopausal women^136,137^. At present, the co-expression and effects of Brn-3b and ER in the heart remain to be investigated and may help to understand the molecular basis for male-female differences in manifestation and outcome of heart disease^136,137^.

**Fig. 5:**
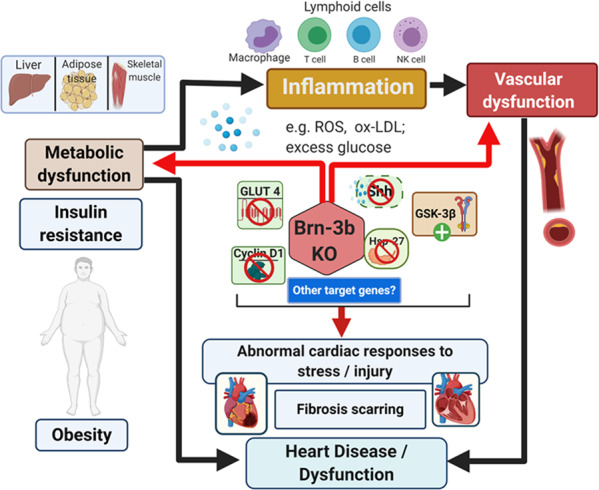
Schematic diagram to show proposed effects by which loss of Brn-3b and its target genes can cause deregulated function in different tissues. Loss of Brn-3b and its target genes may directly or indirectly contribute to adverse effects in insulin-responsive tissue (skeletal muscle, adipose tissue, liver), vascular smooth muscle cells (VSMC) in blood vessels or cardiomyocytes of the heart. Direct effects could arise if the loss of Brn-3b and its target genes are necessary for controlling normal function in specific tissues. In contrast, indirect effects may result from crosstalk between different systemic effects with metabolic dysfunction, caused by loss of Brn-3b giving rise to pro-inflammatory milieu e.g. reactive oxygen species (ROS), oxidative low-density lipoprotein (Ox-LDL), which, in turn, trigger adverse changes in the heart and vasculature.

It is also noteworthy that both cardiac and metabolic diseases are strongly associated with pro-inflammatory responses^5,14^, mediated by immune cells and Brn-3b is highly expressed in monocytes and T lymphocytes that are implicated in such effects^45,46^. Although potential roles for Brn-3b in controlling inflammatory responses are still being investigated, this raises important questions about the inflammatory milieu generated by the loss of Brn-3b and could form part of an interesting interface through which inflammatory responses may drive pathological changes in diverse cell types, and thereby impact on the development of metabolic dysfunction and cardiovascular diseases (Fig. 5).

#### Perspective

This review explores Brn-3b/POU4F2 as a novel regulator of metabolic function and cardiac responses in male hearts, which is based on data from different experimental models and provides compelling evidence to support key roles for this regulator in controlling both metabolic and cardiac responses to stress. While cardiac-specific Brn-3b target genes are still to be identified, the coexistence of metabolic dysfunction and cardiac abnormalities in Brn-3b KO mutants, along with functions of its known target genes in such processes have provided a working model by which loss of this regulator could contribute to cardiometabolic dysfunction and disease (Fig. 5). Since global CVD is predicted to rise in line with an ongoing obesity epidemic, then understanding the molecular mechanisms by which Brn-3b drives diverse effects in different tissues can provide insights into how the loss of Brn-3b contributes to pathological changes in metabolic and cardiovascular diseases, but could also provide different approaches for earlier diagnosis and effective treatment.
